# Contagious itch can be induced in humans but not in rodents

**DOI:** 10.1186/s13041-019-0455-2

**Published:** 2019-04-24

**Authors:** Jing-Shan Lu, Qi-Yu Chen, Si-Bo Zhou, Feng-Yi Wu, Ren-Hao Liu, Zhao-Xiang Zhou, Hua Zhang, Min Zhuo

**Affiliations:** 1 Center for Neuron and Disease, Frontier Institutes of Science and Technology, Xi’an Jiaotong University, Xi’an, 710049 China; 2 Department of Physiology, Faculty of Medicine, University of Toronto, Medical Science Building, Room #3342, 1 King’s College Circle, Toronto, Ontario M5S 1A8 Canada

**Keywords:** Itch, Contagious, Video, Scratch

## Abstract

Itch contagion has been reported in human when people watch someone scratching in a video. The basic mechanism of contagious itch induced by scratching video is still being investigated. A recent study has reported that adult mice showed itch like responses after watching itch-like video or mice showing itching responses. However, such contagious itch behaviors failed to be reproduced by another study by repeating the same experiments of viewing itching mice. It is unclear if contagious itch induced by seeing itching video may be reproducible. In the present study, we used a four-iPad paradigm to repeat these experiments, and found that mice showed no significant itch-like responses after watching itching video of mice. To test if mice actually can see the video, we placed mirrors at the same location. Interestingly, mice showed altered activities in the open field with the mirrors. Finally, in healthy subjects, we found that viewing human itch video did cause itch sensation or responses. Our results indicate that the mouse model may not appropriate for studying contagious itch in humans.

## Introduction

Itch is an irritable sensation that is always described as unpleasant and that provokes scratching behavior. In conventional itch transmission pathways, cutaneous pruriceptors transmit signals through the glutamatergic projection of afferent C-fibers to the spinal dorsal horn [1–3]. Puriceptive information is ascended to the thalamus, and finally processed by itch related cortices, such as the prefrontal cortex, somatosensory cortex, premotor areas, insular cortex, and the anterior cingulate cortex (ACC) [2, 4]. Itch can be relieved by scratching action, which activates spinal interneurons to inhibit itch-transmitting pathway [2, 5]. In addition to mechanical stimuli and some pathological reasons, such as skin diseases and visceral symptoms, itch can also be induced by audiovisual approaches, both in humans and non-human primates. It has been reported that some people may feel itchy when they watch someone else scratching or when they see scenes related to itch. Niemeier et al. [6] found that healthy audiences scratched more when listening to an itch-inducing lecture than listening to a relaxing presentation. Visual cues, like video clips of someone scratching, were found to induce itch sensations in both healthy and atopic dermatitis patients [7], although the itch intensity and frequency in atopic dermatitis patients was higher than that of healthy control. Functional neuroimaging studies have revealed that anterior insular, primary somatosensory, prefrontal and premotor cortices are involved in human contagious itch.

Similar contagious itch has been reported in non-human primates and even in rodents. In adult rhesus macaques, scratching behavior has been recorded when monkeys were placed with a scratching cage mate or shown videos of monkeys scratching [8]. Recently, Yu et al. reported the imitative scratching behavior in mice after the mice watched a conspecific scratching video or observed an actual scratching demonstrator without any prior training or reward [9]. However, those findings failed to be reproduced in a recent study [10]. Furthermore, there is no additional report confirming video-inducing itch in normal mice. Our study aimed at investigating whether contagious itch exists in healthy mice and mice of histamine-inducing itch model when the mice watched videos of other mice scratching. We also observed contagious itch behavior in humans while watching a video of another person scratching.

## Materials and methods

### Animal

Adult, male C57 BL/6J mice aged between 6 and 8 weeks were used in experiments. All mice were housed under a 12 h light/dark cycle with food and water provided ad libitum. Experiments conducted in accordance with protocols approved by the Animal Care and Use Committee of Xi’an Jiaotong University.

### Participants

The participants included 10 healthy adult volunteers. They were made up of 5 men and 5 women, aging from 22 to 30, who were blinded about the design of the experiments. All participants provided written informed consents. The participants completed the behavioral ratings and questionnaires outside of the video-watching room.

### Itching behavior of mice

Mice were handled and habituated for 30 min for 3 days before the experiments. Each mouse was habituated and tested individually in the home cage. Similar with the video paradigm that Yu et al. used [9], one iPad was placed against the transparent walls of the cages. When using four iPads, iPads were placed surrounding the cage. Two cameras were placed at two neighbor sides of the cage between the iPad and the cage wall. The itching demonstrator mouse was chosen randomly and 500 μg histamine (Sigma, USA) dissolved in 50 μl saline was injected subcutaneously to the nape of its neck [1, 4, 11, 12]. The typical scratching activity was recorded and looped on the iPads (Apple, USA). Video of the saline-injected mouse was used as control. For the observers, the same volume of saline or a lower dose of histamine (50 μg) was injected to the nape of necks subcutaneously. The total number of itching bouts of the observers was recorded for 30 min after the injection. A scratching bout was defined as lifting of either hind limb to scratch at the nape of neck and replacing the paw onto the floor, regardless of the number of scratching strokes that occurred between the first lift and final lowering of the hind limb.

### Pain behavior of mice

Mice were handled and habituated for 30 min for 3 days before the experiment in the home cage. Formalin (5%, 10 μl) was injected into the dorsal side of hind paw of the demonstrator mouse [13, 14]. Continuous licking and biting behaviors were recorded and looped on the four iPads. The total number of itching bouts of the observers was recorded for 30 min after injecting 5 μl saline to the plantar of hind paws.

### Mice behavior in the open field with mirror

Mice were placed in a novel open field (43.2 × 43.2 × 30.5 cm^3^) inside a dimly-lit, constant temperature isolation chamber (< 50 lx in the center of the open field). Four mirrors leaned against the walls of the open field. An activity monitoring system (Smart 3.0, Panlab, USA) was used to record horizontal locomotor activity. Briefly, this system uses paired sets of photo beams to detect movement (number of photo beams: 16; space between the beams: 2.5 cm; number of zones: X: 17, Y: 17). Each animal was placed in the center of the open field, and activity was measured for 60 min. Central zone was defined by zones from (4, 4) to (13, 13).

### Human behavior

A man’s performance of continuous scratching his face and chin was recorded and used as the human scratching video. Ten participants were recruited based on their voluntariness. They watched the man’s scratching video individually for 10 min in an isolated room. Their activities and emotions during this period were recorded by cameras without their recognition. After watching the video, participants leaved the room immediately and entered another room to start answering questions as follow:

Please answer the following questions without thinking, tell us your feelings in the first place. Let’s get started.

1. What’s your name?
2. How old are you?
3. Did you feel uncomfortable when you watched the video? If so, please score your discomfort from 0 to 3.
4. Did you feel itching when you watched the video? If so, please score your itch from 0 to 3. If the answer is “yes”, did you scratch during watching the video? How many times?
5. Did you feel anxious when you watched the video? If so, please score your anxiety from 0 to 3.
6. Did you feel amusing when you watched the video? If so, please score your amusement from 0 to 3. If the answer is “yes”, have you laughed?
7. Did you feel painful when you watched the video? If so, please score your pain from 0 to 3.

In addition to verbal answers to these questions, we also recorded the whole video of these participants watching scratching video for 10 min. We recorded the number of scratching and the scratching regions afterwards.

### Statistical analysis

All data are expressed as means ± SEM. For comparison between two groups, unpaired two-tailed *t* test was used. For comparison between three or more groups, one-way ANOVA with post hoc Turkey analysis was performed. *p* < 0.05 was considered statistically significant.

## Results

### Itch video paradigm failed to induce contagious itch in mice

**Fig. 1:**
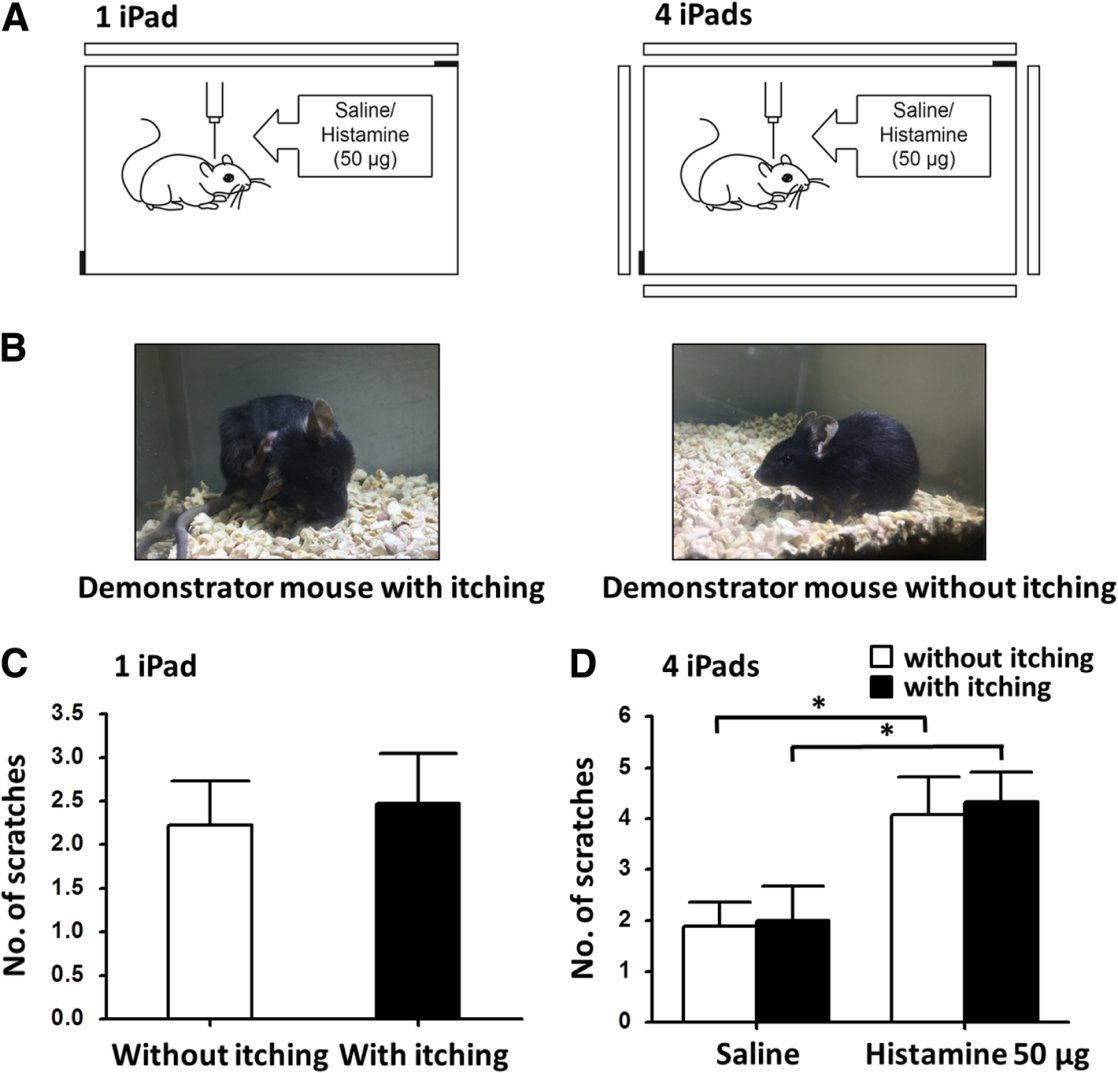
Itch video paradigm failed to induce contagious itch in mice. **a** 50 μl saline (or 50 μg / 50 μl histamine) was injected to the observer mice subcutaneously. The mice were put into the transparent home cage with an iPad (white slab, left) or four iPads surrounding the home cage (right) looping the video of demonstrative mice Two cameras (black rectangles) in the corners recorded the behavior of observer mice in 30 min after injection. **b** Itch-related behavior (Scratching the nape of neck with hind paw, left) and control (right) displayed in the video. **c** In one iPad paradigm**,** the video of itch- related behavior did not induce significant increase of scratching behavior mice in observers in which saline was injected (*p* = 0.737, *n* = 18 for each group, Student’s *t*-test). **d** In four iPads paradigm, the video of itch- related behavior failed to induce significant increase of scratching behavior mice in observers in which saline or lower dose of histamine (50 μg) was injected (*F* = 0.087, *p* = 0.769, n = 18 for each saline-injected group, *n* = 12 for each histamine-injected group, ANOVA). Lower dose of histamine induced enhanced itch in observer mice significantly. *p < 0.05 considered as significant (n = 12 for each group, Student’s *t*-test)

To confirm whether the visual cues were able to induce contagious itch behavior, 50 μl saline was injected to the nape of the neck of the observer mice, which were then placed into individual home cages, identical to the habituation cages, with an iPad on the wall. Videos of the representative behavior of itch or control demonstrator mouse were looped on the iPad (Fig. 1a left, 1B). For the itch demonstrator mice, 500 μg histamine was injected to the nape of neck, which induced intense scratching bouts within 30 min. This dose of histamine indeed induced significant itching responses as compared with saline (mean 61 ± 5 vs. 2 ± 1, *n* = 18, *p* < 0.001). 30 min after saline injection, the total number of itching bouts of the observers showed no significant variance between itch and non-itch video groups (2 ± 1 vs. 2 ± 1, n = 18, *p* > 0.05, Fig. 1c.). Considering that the frequency of looks may be low with single iPad, we used four iPads placed around the cage to play the video simultaneously (Fig. 1a right). Despite all this, demonstrator mouse in the video did not affect the scratching behavior of the observers (2 ± 1 vs. 2 ± 0, n = 18, *p* > 0.05, Fig. 1d.).

To further investigate the effect of the itching video to the observer’s itching behavior, we injected a lower dose of histamine (50 μg) to the nape of the neck of the mice and recorded their behavior in the cage with four iPads looping either the itching or normal video for 30 min. The number of scratching bouts in the histamine injection group was much higher than that of saline injection group (4 ± 1 vs. 2 ± 1, *n* = 12, *p* < 0.01 in scratching video group; 4 ± 1 vs. 2 ± 0, n = 12, *p* < 0.01 in normal video group, Fig. 1d), however the videos did not affect the itching behavior of the observer mice (*F* = 0.087, *p* = 0.769, *n* = 18 for each saline-injected group, *n* = 12 for each histamine-injected group, ANOVA). These results indicate that contagious itch may not exist in rodents in either normal or itching conditions.

### Pain video paradigm failed to induce contagious pain in mice

**Fig. 2:**
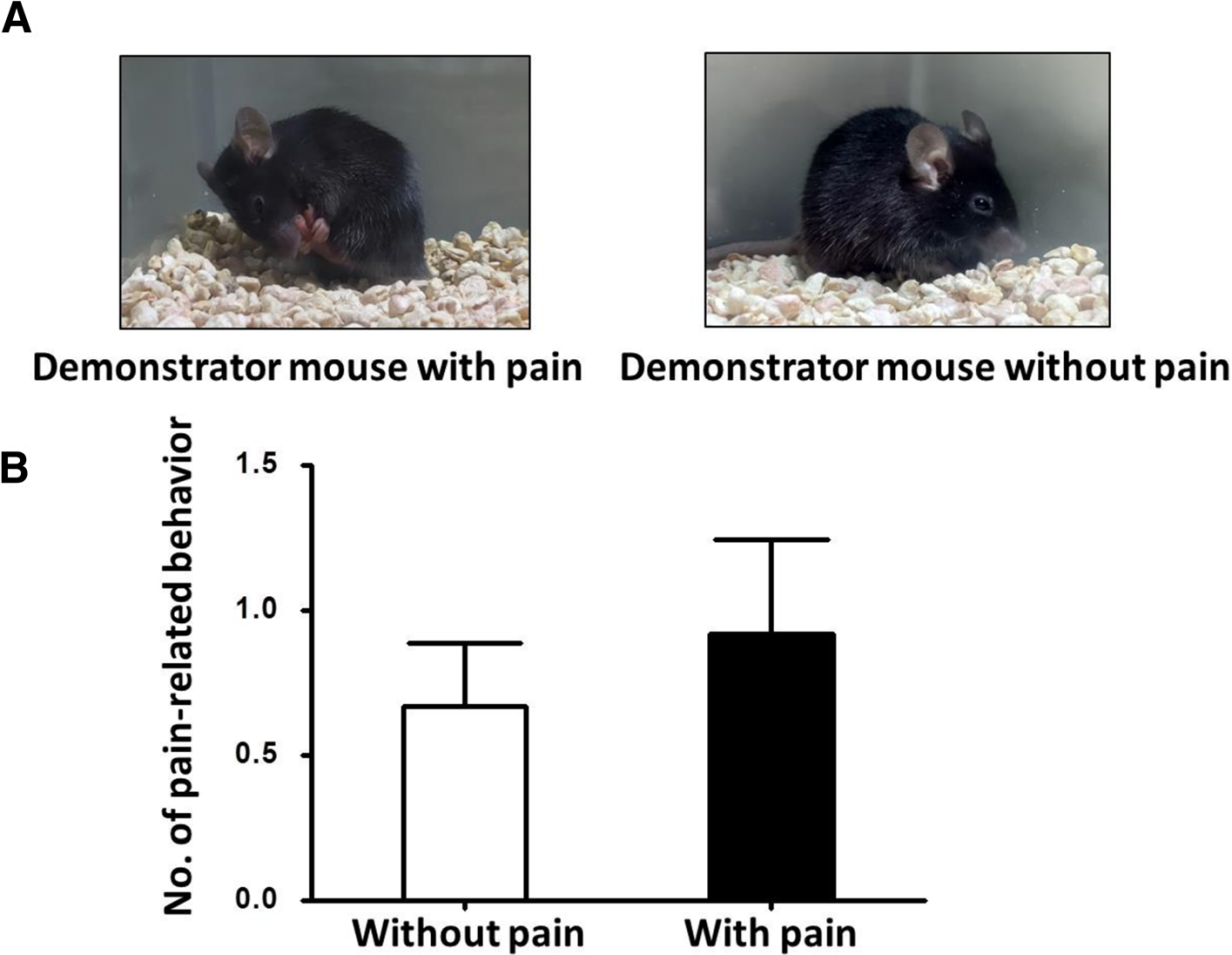
Pain video paradigm failed to induce contagious pain in mice. **a** Pain-related behavior (continuous licking and biting the paw in which formalin was injected, left) and control (right) displayed in the video. **b** In four iPads paradigm, the video of pain-related behavior failed to induce pain related behavior in observer mice compared with those watched normal behavior of demonstrator mouse (*p* = 0.50, n = 12 for each group, Student’s *t*-test)

Using the similar four-iPad paradigm, we observed the pain contagion in mice. The video of continuous licking and biting behavior of the demonstrator mouse failed to induce similar pain related behavior in observers compared with those watched normal behavior of demonstrator mouse (Figs. 2, 1±0 vs. 1±0, *p* = 0.50, n = 12 for each group, Student’s *t*-test). This result indicates pain cannot be transmitted by watching video in rodents.

### Proof of video observation behavior in mice

**Fig. 3:**
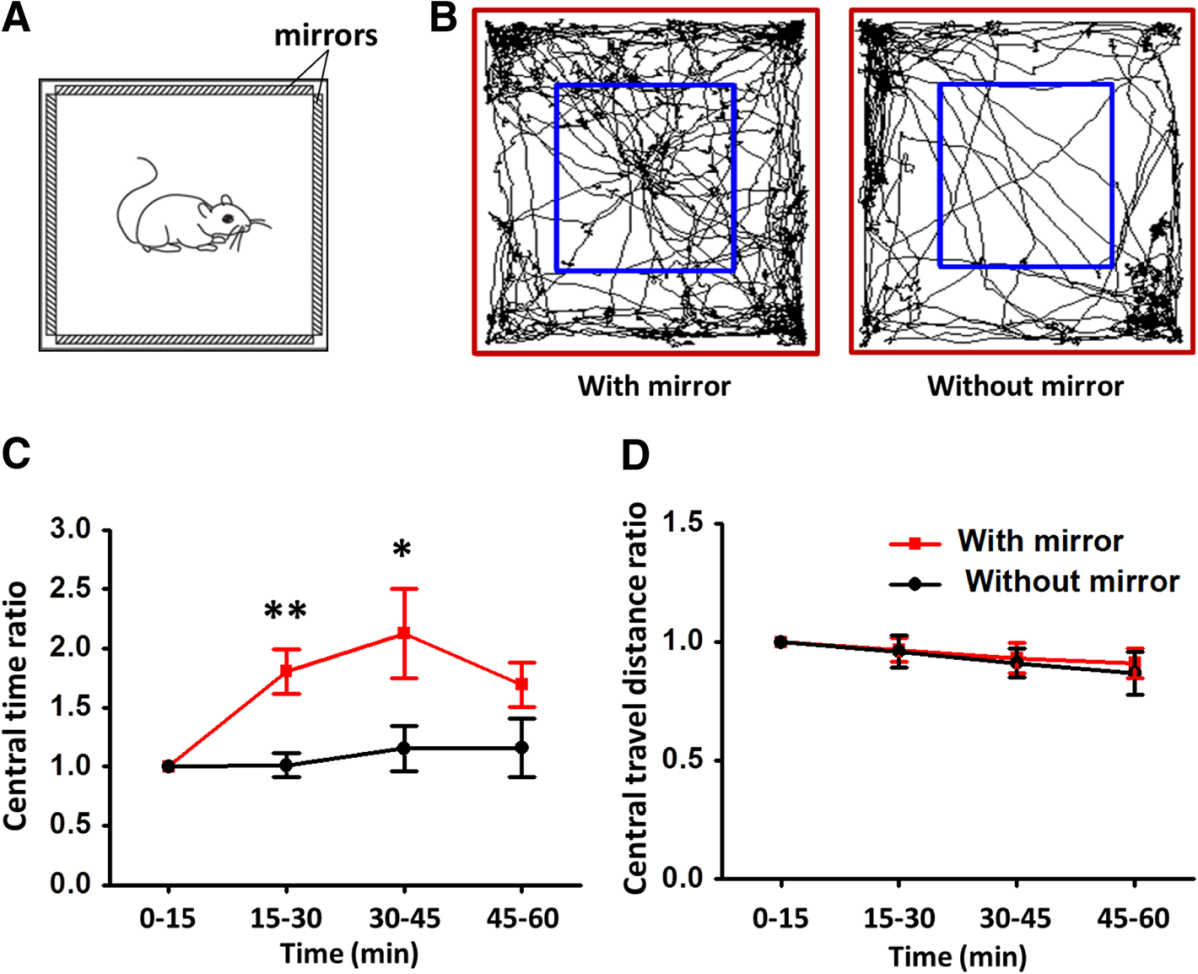
Mirror induced behavior changes in mice. **a** A modified open field with mirrors on the four sides of the inner wall. **b** Representative traces showed the movement of the mice in the open field with (left) and without mirror (right) during 15–30 min after being placed in the open field. The red and blue rectangles represented the border of peripheral and center zones individually. **c** Mice in the open field with mirror spent more time in the central zone during 15–45 min after being placed in the open field compared with those put in the open field without mirror (*p < 0.05, **p < 0.01, *n* = 8 for each group, Student’s *t*-test). The time was displayed as a ratio of the time spent in the central zone with the time spent in the central zone during the first 15 min after being placed in the open field. **d** The travel distance had no significant variance between the open field with mirror and without mirror group

To verify that the videos in the iPads can be seen by the mice and could have effect on the observers, we put mice in an open-field with four mirrors on the same spots with the iPads (Fig. 3a). Mice got close to the mirror and then moved away quickly, and spent more time in the central area compared with those in a normal open field during the 15 to 45 min after being placed in the center of the open field (Fig. 3b and c, 1.81 ± 0.19 vs. 1.01 ± 0.10, **p < 0.01 for 15–30 min and 2.13 ± 0.38 vs. 1.15 ± 0.19, *p < 0.05 for 30–45 min, *n* = 8 for each group, Student’s *t*-test), with no difference in the travel distance (Fig. 3d). These results suggest that mice did see the videos and can be influenced by the demonstrators in the videos.

### Contagious itch was observed in human

We define “contagious itch” in human as viewers reported itch sensation or showed scratching behaviors from similar body areas as shown in the video. To investigate this, we recorded a man’s continuous scratching activity to be used as the demonstrator. Ten participants (aged from 22 to 30, 5 men and 5 women) watched the demonstrator’s scratching video individually for 10 min. The behavior of the observers was recorded and all the observers were asked to describe and score their feeling after watching the video (Table 1). The score of itch was higher (1.4 ± 0.3) than other kinds of senses, such as discomfort (0.8 ± 0.3), anxiety (0.6 ± 0.4), fun (0.7 ± 0.3) and pain (0.1 ± 0.1). Interestingly, most participants scratched (6 ± 3 times) when watching the videos even though some of them did not describe their feeling as itching in the questionnaire, while one participant described her feeling as itchy although she did not scratch (Table 1). Among 10 participants, except for one person didn’t scratch, all the rest nine people did scratch and almost all the scratching activities (58 times in total) concentrated in the head and face areas, but not other areas. In our pre-screening test, all of these individuals do not report itch. Thus, we believe that these scratching activities we recorded is “contagious” or itch caused by viewing the video. These results confirm previous reports that contagious itch does exist in human.

**Table 1:** Summary of human responses to watch the video of itch

No.	Gender	Age	Score of senses: −/+/++/+++					Body Parts	Expression	No. of scratch
			Uncomfortable	Itch	Anxious	Laugh	Pain			
1	♀	22	++	+	–	+	–	above the mouth, nose, eyebrow, face, head	smile	8
2	♂	25	++	+	+	–	–	ear	distracted at last	1
3	♂	22	–	–	–	++	–	above the mouth, middle of eyebrows	smile at first	2
4	♂	29	+	++	+++	–	–	touch face, neck, nape, forehead	frown	3
5	♂	26	–	+	–	–	+	nose (touch the hand twice)	smile at first, sleepy at latter half	1
6	♀	29	–	+	–	++	–	head, face	/	2
7	♀	26	+	+	–	+	–	eyebrow, ear	smile 6 times, yawn 8 times	2
8	♂	25	–	+++	–	+	–	forehead, face, ear, above the mouth, head, hand, eyelid, shoulder, chin, neck	/	35
9	♀	27	–	+	–	–	–	/	/	0
10	♀	30	++	+++	++	–	–	above the mouth, mouth	sleepy at latter half	2

## Discussion

In our study, we successfully observed contagious itching behavior in humans when the healthy candidates watched another man scratching in the video, using the video observing paradigm. However, in adult mice, we failed to observe contagious itch. Furthermore, viewing a video of formalin induced licking behavior also failed to induce licking-like behavior in adult mice. While the result of the human experiment is similar to previous human reports [7], we failed to reproduce video induced contagious itch in mice as reported in a recent study [9].

### Animal models for itching

Histamine is a commonly-used puritogen in animal models of acute itch. Injecting the puritogen into the nape of the neck is a traditional scratching assay in which the number of scratching bouts of the hind limbs was calculated [12, 15]. There are other assays in which puritogens were injected into the cheek, calf and even eyes of the rodents to induce scratching behavior [16–18]. In our study, we used the “nape of neck” assay for the demonstrator mice to induce an acute itch-scratch behavior. We also injected a lower dose of histamine (50 μg) to the observer mice in order to coordinate their experience of itch with the demonstrator mouse. For investigating itch contagion, previous studies have used macaque monkeys and mice [8, 9]. We also tried to induce itch via visual-transmission in humans and mice. However, itch contagion cannot be observed in mice through watching the video of scratching, regardless of being in an itching condition or not.

### Central mechanism for itching

Itch is a commonly-seen negative sensation, which mechanisms have been studied for decades. In terms of itch contagion, brain imaging has proved that watching videos of other people scratching activates the thalamus, primary somatosensory cortex, premotor cortex, and anterior insula of the observers [19–21], which could be the central physiological foundation of the video-transmitted itch.

### Empathy in mice

Empathy is a psychological concept that allows individuals to understand and share the emotions of others. Early studies have identified empathy in rodents [22, 23]. Empathy can be classified as emotional and cognitive subcomponents. The former includes motor mimicry and emotional contagion, which is widely distributed in almost all mammalian. There is no convincing evidence demonstrating that rodents have the latter, which is a higher form of empathy, requiring cognitive processes [24]. Cumulative evidences showed that fear related empathy existed in mice [25, 26]. In addition, pain related empathy occurred when mice displayed more pain-related behavior if they were tested with similarly formalin subcutaneously injected partners [27]. Familiarity is a crucial factor for empathy in the fear and pain contagion. However, when we substitute the actual affective partner with a video, we failed to induce enhanced pain- /itch-related behavior in observer mice, even though our mirror test proved that mice did observe the behavior in the image. This suggests the failure of visual transmission of itch in rodents.

### Failure to reproduce contagious itch in mice

We failed to induce contagious itch via the video of mouse scratching, while Yu et al. succeeded [9]. Liljencrantz et al. commented that contagious itch cannot be observed in normal mice [10]. The response to this comment said that videos were unsuccessful in inducing contagious itch due to a lack of looks to the screen, defined as head movements towards the screen [28]. To avoid this possibility, we designed the four-iPads paradigm, to largely increase the possibility of “look” behavior in mice. We also used mirrors to verify that mice did observe the objects in the iPad screen. Unfortunately, itch contagion has not been observed in the normal observer mice. The increase of the scratching bouts in 50 μg histamine injection group may only due to the puritogenic effect.

### Major physiological difference between humans and animals

The difference between mice and humans in itch contagion can be attributed to their physiological differences in the central nervous system. It is known that the structure and function of human brain cortices are much more complicated than those of rodents. For instance, a potential candidate for the video-transmitted itch in human and non-human primates is the mirror neuron system [29]. Mirror neurons, which were originally discovered in the ventral premotor area of monkeys, fire not only when the animal makes a specific movement, but when it observes the same movement being carried out [29]. Although the actual role of mirror neurons in itch contagion has not yet been elucidated, there are few reports about the mirror neuron system in rodents. Thus, rodents do not seem to be the best animal model to study itch related empathy.

### Future directions and use the appropriate animal models for human diseases

Although the mechanism of contagious itch in humans requires further investigation, the discrepancy of rodent model and human result is that does reveal that rodents cannot always be valid as models for human diseases. More appropriate animal models are suggested to be investigated. For example, tree shrew, a primate-like animal, has powerful potential of being used as primate model for studying high-level cognitive functions due to its higher affinity to humans and more elaborated brain function compared with rodents [30, 31].

## Abbreviation

ACC: anterior cingulate cortex

## References

[1] Koga K, Chen T, Li XY, Descalzi G, Ling J, Gu J, Zhuo M (2011). Glutamate acts as a neurotransmitter for gastrin releasing peptide-sensitive and insensitive itch-related synaptic transmission in mammalian spinal cord. Mol Pain.

[2] Ikoma A, Steinhoff M, Stander S, Yosipovitch G, Schmelz M (2006). The neurobiology of itch. Nat Rev Neurosci.

[3] Han L, Dong X (2014). Itch mechanisms and circuits. Annu Rev Biophys.

[4] Descalzi G, Chen T, Koga K, Li XY, Yamada K, Zhuo M (2013). Cortical GluK1 kainate receptors modulate scratching in adult mice. J Neurochem.

[5] Davidson S, Giesler GJ (2010). The multiple pathways for itch and their interactions with pain. Trends Neurosci.

[6] Niemeier V, Gieler U (2000). Observations during itch-inducing lecture. Dermatology and Psychosomatics/Dermatologie und Psychosomatik.

[7] Papoiu AD, Wang H, Coghill RC, Chan YH, Yosipovitch G (2011). Contagious itch in humans: a study of visual 'transmission' of itch in atopic dermatitis and healthy subjects. Br J Dermatol.

[8] Feneran AN, O'Donnell R, Press A, Yosipovitch G, Cline M, Dugan G, Papoiu AD, Nattkemper LA, Chan YH, Shively CA (2013). Monkey see, monkey do: contagious itch in nonhuman primates. Acta Derm Venereol.

[9] Yu YQ, Barry DM (2017). Molecular and neural basis of contagious itch behavior in mice.

[10] Liljencrantz Jaquette, Pitcher Mark H., Low Lucie A., Bauer Lucy, Bushnell M. Catherine (2017). Comment on “Molecular and neural basis of contagious itch behavior in mice”. Science.

[11] Lee B, Descalzi G, Baek J, Kim JI, Lee HR, Lee K, Kaang BK, Zhuo M (2011). Genetic enhancement of behavioral itch responses in mice lacking phosphoinositide 3-kinase-gamma (PI3Kgamma). Mol Pain.

[12] Kuraishi Y, Nagasawa T, Hayashi K, Satoh M (1995). Scratching behavior induced by pruritogenic but not algesiogenic agents in mice. Eur J Pharmacol.

[13] Wei F, Qiu CS, Kim SJ, Muglia L, Maas JW, Pineda VV, Xu HM, Chen ZF, Storm DR, Muglia LJ, Zhuo M (2002). Genetic elimination of behavioral sensitization in mice lacking calmodulin-stimulated adenylyl cyclases. Neuron.

[14] Shum FW, Wu LJ, Zhao MG, Toyoda H, Xu H, Ren M, Pinaud R, Ko SW, Lee YS, Kaang BK, Zhuo M (2007). Alteration of cingulate long-term plasticity and behavioral sensitization to inflammation by environmental enrichment. Learning & memory (Cold Spring Harbor, NY).

[15] Shimada SG, LaMotte RH (2008). Behavioral differentiation between itch and pain in mouse. Pain.

[16] LaMotte RH, Shain CN, Simone DA, Tsai EF (1991). Neurogenic hyperalgesia: psychophysical studies of underlying mechanisms. J Neurophysiol.

[17] LaMotte RH, Shimada SG, Sikand P (2011). Mouse models of acute, chemical itch and pain in humans. Exp Dermatol.

[18] Laidlaw A, Flecknell P, Rees JL (2002). Production of acute and chronic itch with histamine and contact sensitizers in the mouse and Guinea pig. Exp Dermatol.

[19] Holle H, Warne K, Seth AK, Critchley HD, Ward J (2012). Neural basis of contagious itch and why some people are more prone to it. Proc Natl Acad Sci U S A.

[20] Mochizuki H, Papoiu ADP, Yosipovitch G (2014). Frontiers in neuroscience brain processing of itch and scratching. Itch: mechanisms and treatment. Edited by Carstens E, Akiyama T. Boca Raton (FL): CRC Press/Taylor & Francis (c) 2014 by Taylor & Francis Group, LLC.

[21] Mochizuki H, Baumgartner U, Kamping S, Ruttorf M, Schad LR, Flor H, Kakigi R, Treede RD (2013). Cortico-subcortical activation patterns for itch and pain imagery. Pain.

[22] Church RM (1959). Emotional reactions of rats to the pain of others. J Comp Physiol Psychol.

[23] Rice GE, Gainer P (1962). "altruism" in the albino rat. J Comp Physiol Psychol.

[24] de Waal FBM, Preston SD (2017). Mammalian empathy: behavioural manifestations and neural basis. Nat Rev Neurosci.

[25] Jeon D, Kim S, Chetana M, Jo D, Ruley HE, Lin SY, Rabah D, Kinet JP, Shin HS (2010). Observational fear learning involves affective pain system and Cav1.2 Ca2+ channels in ACC. Nat Neurosci.

[26] Jeon Daejong, Shin Hee-Sup (2011). A Mouse Model for Observational Fear Learning and the Empathetic Response. Current Protocols in Neuroscience.

[27] Langford DJ, Crager SE, Shehzad Z, Smith SB, Sotocinal SG, Levenstadt JS, Chanda ML, Levitin DJ, Mogil JS (2006). Social modulation of pain as evidence for empathy in mice. Science (New York, NY).

[28] Barry DM, Yu YQ (2017). Response to comment on "molecular and neural basis of contagious itch behavior in mice".

[29] Iacoboni M (2009). Imitation, empathy, and mirror neurons. Annu Rev Psychol.

[30] Lu JS, Yue F, Liu X, Chen T, Zhuo M (2016). Characterization of the anterior cingulate cortex in adult tree shrew. Mol Pain.

[31] Li XH, Song Q, Chen QY, Lu JS, Chen T, Zhuo M (2017). Characterization of excitatory synaptic transmission in the anterior cingulate cortex of adult tree shrew. Molecular brain.

